# En Face Choroidal Vascularity in Both Eyes of Patients with Unilateral Central Serous Chorioretinopathy

**DOI:** 10.3390/jcm12010150

**Published:** 2022-12-24

**Authors:** Filippo Tatti, Claudio Iovino, Giuseppe Demarinis, Emanuele Siotto Pintor, Marco Pellegrini, Oliver Beale, Kiran Kumar Vupparaboina, Mohammed Abdul Rasheed, Giuseppe Giannaccare, Jay Chhablani, Enrico Peiretti

**Affiliations:** 1 Eye Clinic, Department of Surgical Sciences, University of Cagliari, 09124 Cagliari, Italy; 2 Eye Clinic, Multidisciplinary Department of Medical, Surgical and Dental Sciences, University of Campania Luigi Vanvitelli, 80131 Naples, Italy; 3 Ophthalmology Unit, S. Orsola-Malpighi University Hospital, University of Bologna, 40138 Bologna, Italy; 4 Department of Ophthalmology, University of Pittsburgh, Pittsburgh, PA 15213, USA; 5 School of Optometry and Vision Science, University of Waterloo, Waterloo, ON N2L3G1, Canada; 6 Department of Ophthalmology, University “Magna Graecia”, 88100 Catanzaro, Italy

**Keywords:** central serous chorioretinopathy, pachychoroid, en face optical coherence tomography, choroid, choroidal vascularity index

## Abstract

The aim of this study was to evaluate the choroidal vascularity analyzing en face optical coherence tomography (OCT) images in patients with unilateral central serous chorioretinopathy (CSC). We retrospectively evaluated 40 eyes of 20 CSC patients and 20 eyes of 10 gender- and age-matched healthy individuals. The sample consisted of: (1) CSC affected eyes; (2) unaffected fellow eyes; (3) healthy eyes. Multiple cross-sectional enhanced depth imaging OCT scans were obtained to create a volume scan. En face scans of the whole choroid were obtained at 5μm intervals and were binarized to calculate the choroidal vascularity index (CVI). The latter, defined as the proportion of the luminal area to the total choroidal area, was calculated at the level of choriocapillaris, superficial, medium and deep layers. No significant differences between choriocapillaris, superficial, medium and deep CVI were found in both eyes of CSC patients, whereas a significant different trend of changes was found in healthy eyes. Nevertheless, the en face CVI shows no difference between affected fellow and healthy eyes. In conclusion, CSC-affected eyes and fellow eyes showed a similar vascular architecture, with no statistical difference between all choroidal layers.

## 1. Introduction

Central serous chorioretinopathy (CSC) is characterized by localized serous detachment of the neurosensory retina, with or without focal detachments or alterations of the retinal pigment epithelium (RPE) [1,2]. This disorder, mostly seen in young and middle-aged males, typically is self-limited, but it may recur or persist in the chronic form of the disease [1]. Although CSC usually manifests in one eye, it may occur as a bilateral condition. Under this light, in the literature, the incidence of bilateral CSC at the initial visit is reported to be between 5% to 18% [3], whereas a bilateral involvement was found to increase with a longer follow-up [3,4,5,6].

The alteration of the choroidal vasculature is a well-known factor in the pathogenesis of CSC [7]. The choroidal involvement was firstly demonstrated by the features on an indocyanine angiography (ICGA), such as hyperpermeable dilated choroidal vessels [8], and this is considered a hallmark of the disease. However, while the ICGA is able to better delineate the choroidal vessels, it does not allow to localize the vascular features in their respective tissue layers [9,10,11]. Therefore the optical coherence tomography (OCT) development and the introduction of novel imaging techniques, such as enhanced-depth imaging (EDI) and swept source (SS), have facilitated the detailed and depth-resolved evaluation of the choroidal morphology in CSC patients [12,13,14].

Additionally, a choroidal vasculature evaluation in CSC patients was obtained using the choroidal vascularity index (CVI), a new parameter defined as the ratio between the luminal choroidal area (LCA) and the total choroidal area (TCA) on OCT B-scans [15,16,17,18]. In a recent study, this parameter allowed to show an increased vascular component compared with the stromal component in eyes affected by CSC. Indeed, an increased choroidal vascularity index was demonstrated in affected eyes compared with fellow ones. However, fellow eyes also showed a higher CVI in comparison with age-matched healthy subjects. As previously reported, the CVI could then be a useful index for early diagnosis of CSC and the assessment of the treatment response after photodynamic therapy [16,17].

Nevertheless, the CVI measured on the foveal cross-sectional B-scan cannot reveal the overall picture of the choroidal status [19,20,21]. For this reason, the CVI has been recently measured also on en face OCT scans to obtain a more real representation of the choroidal vasculature in healthy or affected eyes [22,23]. The en face CVI evaluation at various levels of the choroid showed a similar trend of changes in acute and chronic CSC patients [23].

The aim of the present study was to evaluate the CVI changes in both eyes of patients with unilateral CSC by analyzing en face OCT images generated through volumetric maps.

## 2. Materials and Methods

A consecutive series of 20 patients with diagnoses of unilateral CSC were evaluated in this retrospective study. All subjects were attended to at the Retina Center of the Eye Clinic, University of Cagliari. The study adhered to the tenets of the Declaration of Helsinki and the protocol used was approved by the local Institutional Review Board (NP/2022/3119). A complete ophthalmic examination was performed for each patient, including Snellen best-corrected visual acuity (BCVA), fundus autofluorescence, fluorescein angiography (FA) and ICGA (Heidelberg Spectralis, Heidelberg Engineering), intraocular pressure (IOP) measurement, anterior segment and fundus examination. Unilateral CSC was defined as a prior or active unilateral manifestation of CSC. Thus, patients evidencing any presence or evidence of previous subretinal fluid in the fellow eyes were excluded from the study. The exclusion criteria were also refractive error >±3, macular pathologies other than CSC, as well as the presence of MNV and any ocular surgery. Patients with a history of any treatment in the previous 3 months and of any previous treatment that could affect CVI were also excluded [24,25]. A history of any previous medications that could cause subretinal fluid was also recorded. The patient group was compared with a gender- and age-matched control group (20 eyes of 10 healthy individuals).

### 2.1. Spectral-Domain Optical Coherence Tomography Analysis

For each eye, a posterior pole volumetric scan containing multiple high density cross-sectional scans (49B, 30 × 20°) was obtained using the spectral-domain (SD) OCT with EDI mode. The scans were obtained for each patient in the afternoon at the set time frame 2–4 pm. These data were exported from the Heidelberg device as images with a 1:1 pixel ratio.

Central macular thickness (CMT) was defined as the average thickness of a 1 mm diameter circle centered on the foveal center, measuring from the internal limiting membrane and the RPE. Subfoveal choroidal thickness (CT) was obtained by measuring the distance between RPE–Bruch’s membrane complex and the choroidoscleral interface.

### 2.2. Choroidal En Face OCT Extraction

The algorithm involved in obtaining the en face CVI measurement included the choroidal en face OCT extraction and the binarization of the en face OCT scans, following an already tested procedure [22].

The choroid was firstly segmented from the OCT volume. In particular, each B-scan of the volume scan was analyzed to segment choroid on a previously validated algorithm where the RPE–Bruch’s membrane complex and the CSI were identified using structural similarity (SSIM), Hessian analysis and tensor voting [26]. Segmented choroidal sections were subsequently stacked to obtain the choroid volume, and multiple 5 micron spacing en face sections were generated for the CVI analysis.

### 2.3. En Face CVI estimation

Adaptive histogram equalization was employed (using a built in MATLAB v2018b function) in order to increase the contrast between choroidal vessel lumen and the stroma. Blood vessels were then separated using the block-based particle swarm optimization (PSO) thresholding [22,27]. The binarized images were reviewed by two independent observers blinded to each other to assess whether the images were correctly converted by comparing with the original en face OCT images. This process was performed twice for each image by each observer.

CVI was calculated for every en face image separated by 5 μm within the choroid volume. The layer of small choroidal vessels, including choriocapillaris, was defined as a dense network of small vessels just 10 μm beneath Bruch’s membrane.

**Figure 1:**
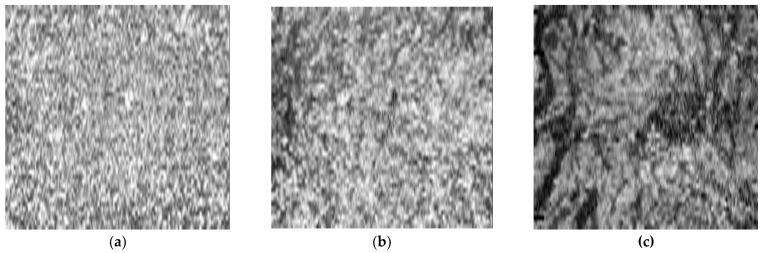

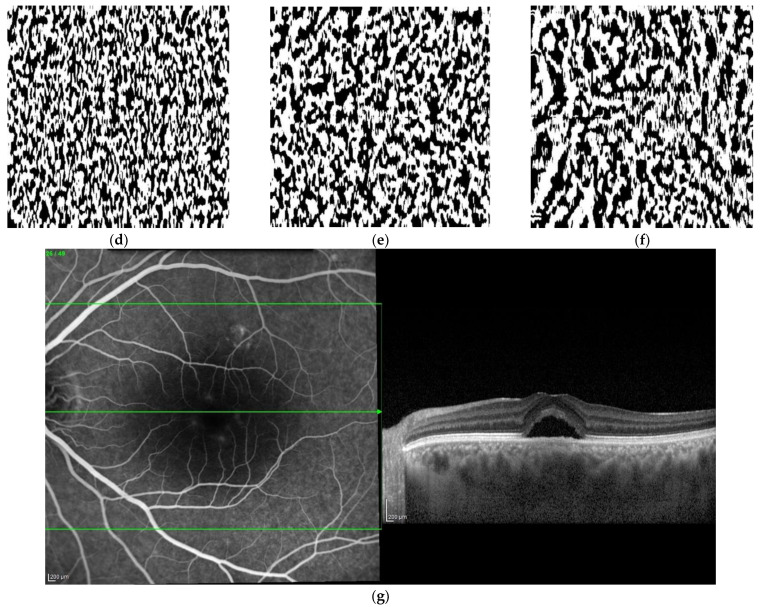
Original en face optical coherence tomography (OCT) scans and the software-processed images of an affected eye of a patient with central serous chorioretinopathy. Original en face OCT scan images of the superficial (**a**), medium (**b**) and deep (**c**) choroidal layer; binarized images of the superficial (**d**), medium (**e**) and deep (**f**) choroidal layer; OCT B-scan across the foveal center (**g**).

**Figure 2:**
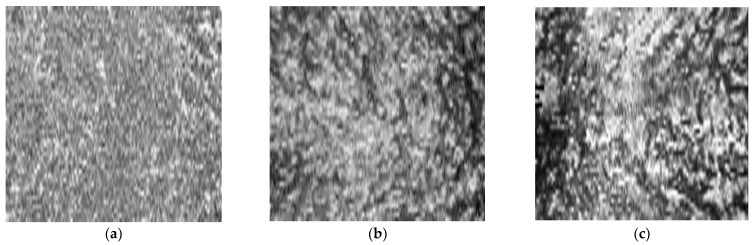

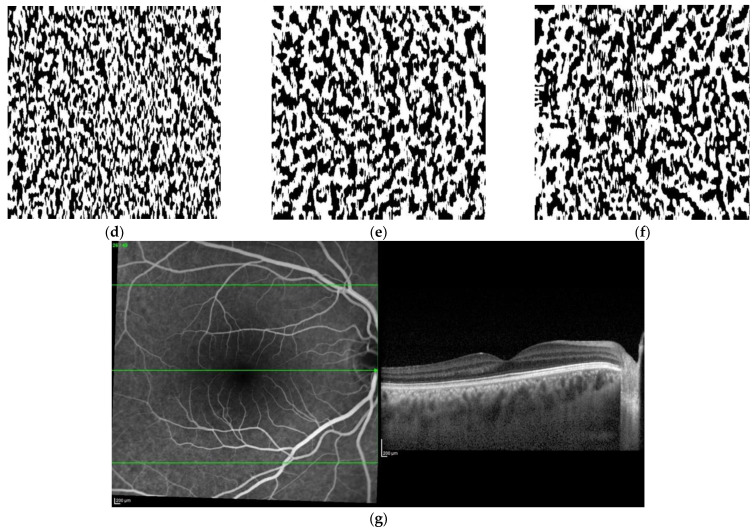
Original en face optical coherence tomography (OCT) scans and the software-processed images of the fellow eye of the same patient of Figure 1. Original en face OCT scan images of the superficial (**a**), medium (**b**) and deep (**c**) choroidal layer; binarized images of the superficial (**d**), medium (**e**) and deep (**f**) choroidal layer; OCT B-scan across the foveal center (**g**).

The points of measurements were manually identified in each eye, focusing on major anatomical locations (i.e., Bruch’s membrane, choriocapillaris and choroidoscleral interface) and at various depths from RPE–Bruch’s membrane complex. The maximum choroidal thickness across the volume cube was divided by three (superficial or inner layer, medium and deep or outer layer) for both eyes. Hence, the mean CVI was calculated for the choriocapillaris, the inner/superficial third, the middle/medium third and the outer/deep third of the choroidal thickness. (Figure 1 and Figure 2).

### 2.4. Statistical Analysis

The statistical analysis was conducted with R (version 4.0.0) and RStudio (version 1.2.5042) software. The Kolmogorov–Smirnov test was used to evaluate the normal distribution for each variable. The CVI was compared between CSC eyes and fellow eyes by using paired samples t-test or Wilcoxon test. A repeated measures ANOVA or Friedman test was used to compare the choroidal vascularity of choriocapillaris, superficial, medium and deep third of the choroid. A *p* value < 0.05 was considered statistically significant.

## 3. Results

A total of 20 patients (16 males and 4 females) were included. The average age was 50.7 ± 9.96 years. The average BCVA was 0.28 ± 0.35 logMAR for CSC eyes and 0.03 ± 0.09 logMAR for fellow eyes. Previous treatments included only nonsteroidal anti-inflammatory drugs (10 patients). The average time between the diagnosis and the evaluation was 2.42 ± 2.47 years.

The gender- and age-matched control group included 20 eyes of 10 individuals (eight males and two females) with a mean age of 48.8 ± 3.5 years. The demographic data showed no statistical difference with the study group (all *p* > 0.05).

The choroidal parameters in CSC, fellow and healthy eyes are reported in Table 1.

The subfoveal CT was significantly higher in eyes with CSC compared with fellow eyes (489.8 ± 13.4 vs. 433.7 ± 12.2; *p* = 0.047). The first third segment thickness resulted on average 163.3 ± 44.8 μm and 144.6 ± 41.6 μm for affected eyes and fellow eyes, respectively. Consecutively, these values represented the average thicknesses of the choroidal segments. For the average en face CVI, no significant difference between the CSC and fellow eyes was observed (*p* = 0.681). Similarly, no significant differences in the choriocapillaris, superficial, medium and deep CVI were found (respectively, *p* = 0.940, *p* = 0.685 and *p* = 0.411; *p* = 0.627) (Table 1).

There was a significant difference in subfoveal CT between healthy eyes and both eyes of CSC patients. However, with regard to the CVI layers’ comparison, no difference was shown in the layer comparison between healthy and CSC or fellow eyes.

Although a different trend of changes between CSC eyes and fellow eyes, choriocapillaris, superficial, medium and deep CVI did not significantly differ for both (*p* = 0.73; *p* = 0.16). On the contrary, healthy eyes showed a significant difference of CVI among the various choroidal layers (*p* < 0.01).

## 4. Discussion

We studied the CVI changes across the entire depth of the choroid in both eyes of patients affected by unilateral CSC. In the CSC eyes, the CVI increased as the distance from RPE increased to reach a peak (0.500) in the medium depth of choroid and then reduced towards the CSI (0.495). On the contrary, the mean CVI of fellow eyes tended to reduce from RPE to the CSI (0.501; 0.493; 0.467). The control group showed a different trend, with the lowest average vascular density in the medium layer (0.506; 0.492; 0.506).

Previous studies analyzed the CVI changes in healthy eyes, showing the highest average vascular density in the outer level or Haller’s layer [19,20,21,22]. Sohrab et al. analyzed only three choroidal sections of en face scans and calculated the vessel density on the basis of a preselected threshold of red, green and blue (RGB) intensity. The authors showed a different average vascular density in choriocapillaris (76.5%), Sattler’s layer (83.6%) and Haller’s layer (87.2%) [19].

In another study with a cohort of 30 healthy eyes, the CVI values were 53.16%, 51.38% and 55.69%, respectively, at the level of choriocapillaris, medium choroidal vessel and large choroidal vessel layers [22].

The en face CVI of patients affected by acute or chronic CSC was noted to increase as the distance from Bruch’s membrane increased. Patients with acute CSC had the point of maximum vascularity (48.35% ± 2.06%) at 75% depth of CT, while those with chronic CSC reached the peak vascularity level at 50% of the choroidal depth, with a CVI of 48.70% ± 1.32% [23].

In our cohort, the variation in CVI between choriocapillaris, superficial, medium and deep level of the choroid were not significant for both eyes. These results are in contrast to those previously reported [23]; a possible reason is the different choroidal segmentation method applied. Indeed, Wong et al. compared choriocapillaris and various choroidal depths of CVI (25%, 33%, 50%, and 75%). Moreover, we have observed no significant difference between the whole and various CVI layers of CSC and fellow eyes, which suggests a similar vascular architecture in both affected and fellow eyes. This could support the theory of a bilateral involvement of CSC, previously revealed by many studies [3,4,5,6,8,28,29,30,31] and found to increase with a longer follow-up [3,4,5,6].

Another aspect to consider is that CSC, as a pachychoroid condition [32], is characterized by an increase in the size of Haller’s vessels, which may compress the inner layers and determine a similar vascularity throughout the CT [32,33]. In fact, in severe cases the choriocapillaris and intermediate caliber vessels could be so attenuated that the Haller’s layer would occupy a significant proportion of CT [32].

Choroidal thickness analysis suggests that choroidal thickness in eyes with CSC is larger than that in age-matched control eyes and fellow eyes [30,31,34,35]. Considering the multiple factors that could influence the choroidal thickness (age, axial length, refractive error, blood pressure, time of the day), there is no definitive threshold for defining an eye as having pachychoroid [32]. Nevertheless, according to a previous study that considered 395 µm as a sensitive value to diagnose the “pachychoroid” disease, subfoveal CTs of affected and fellow eyes were increased [36].

Interestingly, our study shows how the choroid plays an important role in the pathogenesis of CSC but that it is not the only player. In fact, other than the similar vasculature and the pathological choroidal thickness, there were no signs of CSC in the fellow eye group. In this respect, it is recognized that other factors, such as the RPE, could play defensive roles against high choroidal hydrostatic pressure [37].

The strength of our study is that we provided a measure in vivo of the vascularity across the depth of the choroid, showing some similarities and differences between study eyes and fellow eyes of patients affected by CSC. The study had several weaknesses. First, was the small sample size; indeed, the strict criteria for unilaterality of the disease led to the exclusion of many cases. Considering this limitation, the research should be considered as just a preliminary study that could not provide any definite conclusion. Second, the single time measuring of CVI does not take account of the choroidal variations based on blood pressure and time of day [15]. Lastly, two further limitations arise from the arbitrary cut-offs in identifying the choriocapillaris and the manual identification of the other points of measurement.

## 5. Conclusions

In this preliminary study, the en face CVI of both eyes of patients affected by CSC showed no difference between affected and fellow eyes. The trend of changes in CVI for CSC and fellow eyes showed no statistical difference in the choroidal layer comparison. On the contrary, healthy eyes showed a significant difference in CVI across the depth of the choroid.

## Figures and Tables

**Table 1:** Choroidal parameters in eyes with CSC, fellow and healthy eyes.

Parameter (Mean ± SD)	CSC Eyes (20)	Fellow Eyes (20)	CSC vs. Fellow Eyes (*p*)	Healthy Eyes (20)	CSC vs. Healthy Eyes (*p*)	Fellow vs. Healthy Eyes (*p*)
Subfoveal CT (μm)	489.8 ± 13.4	433.7 ± 12.2	0.047	334 ± 58.2	<0.01	<0.01
Whole CVI	0.494 ± 0.045	0.484 ± 0.044	0.681	0.488 ± 0.002	0.372	0.955
Choriocapillaris CVI	0.491 ± 0,082	0.497 ± 0.060	0.940	0.469 ± 0.004	0.704	0.900
Superficial layer CVI	0.497 ± 0.020	0.500 ± 0.024	0.685	0.506 ± 0,001	0.690	0.273
Medium layer CVI	0.498 ± 0.027	0.490 ± 0.021	0.411	0.492 ± 0.004	0.088	0.370
Deep layer CVI	0.487 ± 0.107	0.463 ± 0.103	0.627	0.506 ± 0.010	0.448	0.081
Choroidal layer comparison (*p*)	0.73	0.16		<0.01		

CSC—central serous chorioretinopathy; CT—choroidal thickness; CVI—choroidal vascularity index.

## Data Availability

Not applicable.
